# Development of Nonalcoholic Hepatopathy: Contributions of Oxidative Stress and Advanced Glycation End Products

**DOI:** 10.3390/ijms141019846

**Published:** 2013-10-01

**Authors:** Juliana Célia de F. Santos, Iara B. Valentim, Orlando R. P. de Araújo, Terezinha da R. Ataide, Marília O. F. Goulart

**Affiliations:** 1 Institute of Chemistry and Biotechnology, Federal University of Alagoas (IQB/UFAL), Maceio, Alagoas 57072-900, Brazil; E-Mails: jcfsnut@hotmail.com (J.C.F.S.); ibvalentim@yahoo.com.br (I.B.V.); orlando_rpa@hotmail.com (O.R.P.A.); 2 Federal Institute for Education, Science and Technology of Alagoas, Maceio, Alagoas 57020-600, Brazil; 3 Faculty of Nutrition, Federal University of Alagoas (FANUT/UFAL), Maceio, Alagoas 57072-970, Brazil; E-Mail: terezinhadarochaataide@gmail.com; 4 Northeast Biotechnology Network (RENORBIO), Federal University of Alagoas (UFAL), Maceio, Alagoas 57072-900, Brazil

**Keywords:** oxidative stress, liver, AGEs, nonalcoholic fatty liver disease, molecular mechanism of biological activity

## Abstract

Advanced glycation end products (AGEs) are generated spontaneously in cells; however, under conditions of hyperglycemia and lipid peroxidation, their levels are higher than usual, which contribute to the development of diseases such as the nonalcoholic fatty liver disease (NAFLD). NAFLD is associated with oxidative stress (OS), which is linked to the transition of steatosis to steatohepatitis due to lipid peroxidation. The AGE-receptor interaction in hepatic stellate cells leads to an increase in reactive oxygen species and enhances the proliferation and activation of these cells, worsening liver fibrosis and disease progression. In this vicious cycle, there is production of (carboxymethyl)lysine, a biomarker for products of advanced glycation and lipid peroxidation, being a shared component between the two pathways. In this review, we aim to compile evidence to support the basic molecular mechanisms of AGEs and OS generation and their influence, independently or combined, on the evolution of NAFLD. The deeper understanding of the interrelations of AGEs + OS may help to elucidate the pathogenic pathways of NAFLD and to devise rational therapeutic interventions for this disease, with an expected positive impact on quality of life of patients.

## 1. Introduction

The liver has a pivotal role in several metabolic processes, including carbohydrate and lipid metabolism, contributing to plasma protein synthesis, hormone production, along with detoxification of deleterious substances. The liver is unique in its ability to undergo compensatory hyperplasia after cell loss. The hepatocytes perform the majority of functions, while LECS (liver sinusoidal endothelial cells), Kupffer cells and hepatic stellate cells (HSCs) play different roles. LECS regulate diffusion between blood and surface of hepatocytes, Kupffer cells are macrophages, responsible to secrete potent mediators of the early inflammatory response, such as reactive oxygen species (ROS) and HSCs store vitamin A, control the turnover of the extracellular matrix and regulate the contractibility of sinusoids [1].

Livers generate low levels of ROS, especially anion radical superoxide (O_2_^•−^), in the mitochondria, and hydrogen peroxide (H_2_O_2_), as a normal function of various oxidases. Each hepatocyte expresses superoxide dismutases (SOD1 in the cytosol, SOD2 in mitochondria), glutathione peroxidases (cytosol and mitochondria), catalase (peroxysomes), thioredoxins (Trx1 in cytosol, Trx2 in mitochondria) and peroxyredoxins (Prx-I, -II, -VI in the cytosol, Prx-III, -V in mitochondria). It also contains glutathione in mM concentrations in all cellular compartments, has radical chain-breaking antioxidants (vitamin E) in cell membranes, and keeps redox-active iron tightly bound to storage or transport proteins [2].

**Figure 1:**
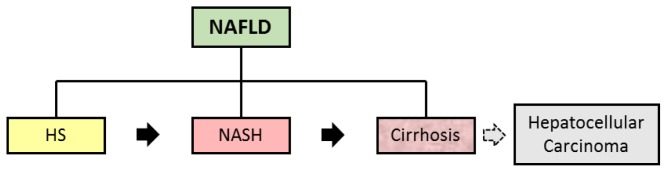
NAFLD Components. The NAFLD is composed by HS, NASH and cirrhosis, which can progress to hepatocellular carcinoma. NAFLD, nonalcoholic fatty liver disease; HS, hepatic steatosis; NASH, nonalcoholic steatohepatitis.

Hepatic Steatosis (HS) is a growing clinical problem worldwide. It is associated with obesity, insulin resistance (IR) (reduced tissue sensitivity to insulin action), diabetes mellitus (DM) and metabolic syndrome (MS) and is one of the alterations that characterize nonalcoholic fatty liver disease (NAFLD). NAFLD is histologically further categorized into HS and nonalcoholic steatohepatitis (NASH). HS is characterized by the presence of hepatic fat accumulation with no evidence of hepatocellular injury in the form of ballooning of the hepatocytes, while NASH by the presence of HS and inflammation with hepatocyte injury (ballooning) with or without fibrosis (Figure 1, Table 1) [3–5].

Advanced glycation end products (AGEs) are a heterogeneous group of molecules produced by *in vivo* glycation and oxidation [11,12]. Glycation is the main spontaneous cause of cellular and extracellular protein damage in physiological systems. Its formation occurs slowly under physiological conditions, however under conditions of IR with hyperglycemia and lipid peroxidation (LP), generation of AGEs increases considerably, contributing to the development of chronic diseases such as DM, atherosclerosis, Alzheimer’s disease, renal failure and gastrointestinal disorders such as HS and hepatic cirrhosis [13–17].

AGEs modify chemical and biological properties of several molecules by cross-linking with these molecules or by interacting with matrix proteins and specialized receptors such as the receptors for advanced glycation end products (RAGE) [10,11,18]. Depending on the cell type and experimental conditions, the AGEs/RAGE interaction in hepatocytes and hepatic stellate cells leads to an increased production of reactive oxygen species through activation of nicotinamide adenine dinucleotide phosphate (NADPH) oxidase. This enhances cell proliferation and activation, thus playing a role in the progression of hepatic fibrosis to NASH [10,19].

Another mechanism likely involved in the progression of NAFLD to NASH is LP, induced by oxidative stress (OS). OS may be defined as a state of imbalance between the factors that generate ROS and those that protect the cell (the antioxidant system) [9], leading to structural alterations of biomolecules (DNA, carbohydrates, proteins and lipids), loss of cell signaling and gene expression control, apoptosis or necrosis [20–22]. The antioxidant systems present in human blood or other biological fluids are divided into enzymatic (superoxide dismutase, catalase, glutathione peroxidase and reductase) and non-enzymatic (glutathione and other thiols, tocopherols, ascorbate, uric acid and β-carotene, transferrin and ceruloplasmin, among others) [21–24]. In OS, the antioxidant system may be deficient and chemical changes in essential biomolecules due to ROS are the main cause for the functional inactivation of these molecules or for their programmed cell death, apoptosis, or even for cellular adaptive response with activation of redox-sensitive transcription factors (e.g., nuclear factors NF-κB (nuclear factor kappa B), Nrf-1 (nuclear respiratory factor 1) and Sp-1 (specificity protein 1) which contribute to the production of proinflammatory and fibrogenic mediators mediated by Kupffer cells and HSCs [25].

In NASH, an increase in cytochrome P450 2E1 (CYP2E1) expression, results in a significant increase in the levels of ROS, with subsequent LP and formation of malondialdehyde (MDA) and 4-hydroxynonenal. Besides CYP2E1, the stress in the endoplasmic reticulum and the stages of redox-sensitive inflammatory signaling are also associated with disease progression. NAFLD emerges as the main cause for chronic liver disease in association with the increased prevalence of obesity and type 2 diabetes in the population. OS and IR are the major contributors to NAFLD pathogenesis and to the progression of steatosis to steatohepatitis [26,27].

The present review compiles evidence to support the basic molecular mechanisms of AGEs and OS generation and their influence, independently or combined, in the evolution of hepatic disease without complications, HS, to advanced pathophysiological process, especially NASH. The deeper understanding of the interrelations of AGEs + OS may help on the elucidation of the pathogenic pathways of NAFLD and to devise rational therapeutic interventions for this disease, improving expectancy and quality of life of patients.

## 2. NAFLD, IR and MS

In normal liver tissue, the content of triglycerides (TG) in hepatic cells is regulated by the integrated activity of cellular molecules that facilitate TG entry in the liver. The synthesis and esterification of fatty acids (FFA) are called input, and fatty acid oxidation and hepatic TG exportation through very low density lipoproteins (VLDL) are called output. Steatosis occurs when the input exceeds the output capacity [3,4,23]. Steatosis may be associated with alcohol abuse, obesity, excessive nutrition support, total parenteral nutrition, hepatitis induced by drugs or toxins, type 2 diabetes, cachexia and postoperative of patients undergoing jejuno-ileal derivation [28]. Approximately 3% of steatohepatitis cases progress to more advanced stages of NAFLD such as fibrosis, cirrhosis, hepatocellular carcinoma and necrosis (Figure 1). The fatty liver until recently was considered a benign condition where only physical exercise and weight loss could contribute to the reversal of the condition. However, currently there is consensus that liver fat accumulation sensitizes the body to develop other types of chronic diseases [5,29].

**Figure 2:**
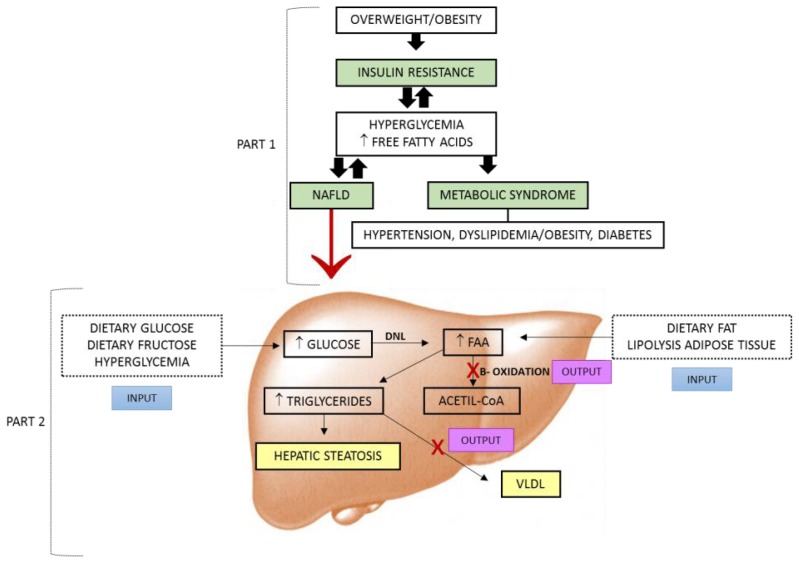
Development of hepatic steatosis and its association with metabolic syndrome (MS). **Part 1**: Overweight/obesity leads extra-hepatic tissues to insulin resistance (IR), resulting in hyperglycemia and increased influx of FFA into the blood, common factors in the development of key components of MS (hypertension, obesity/dyslipidemia, diabetes mellitus (DM)), similar to NAFLD. NAFLD also increases glucose levels and release of FFA in the blood, contributing to the maintenance of IR; **Part 2**: Input-excess dietary glucose/fructose and hyperglycemia, increased lipid synthesis via DNL and increased dietary fat and adipose tissue lipolysis exacerbating IR, increased lipogenesis. Output corresponds to oxidative mitochondrial dysfunction and production of VLDL. Hepatic steatosis in both processes is impaired in function. NAFLD: nonalcoholic fatty liver disease; Input: synthesis and esterification of fatty acids; FFA: free fatty acids; DNL: *de novo* lipogenesis; Output: fatty acid oxidation and hepatic triglycerides exportation; VLDL: very low density lipoproteins.

The estimated worldwide prevalence of NAFLD based on several evaluation methods ranges from 6.3 to 33% of the population, with an average of 20%. The main risk factors for the disease are obesity, type 2 DM and dyslipidemia [5]. NAFLD typically occurs in patients with no history of significant alcohol consumption and is closely associated with abdominal obesity, atherogenic dyslipidemia, hypertension, IR and glucose intolerance, cardiovascular risk factors, all components of MS (Figure 2, Table 1) [30]. For NASH, lower prevalence values are observed, ranging from 3% to 5%. There are no data available for cirrhosis caused by NASH. Further studies are needed to understand the incidence of NAFLD in different age, ethnic and geographic groups [5].

NAFLD is not a diagnosis component of MS, however, like the other diseases that are part of the syndrome, it has IR associated to its development. An important observation shown by scientific studies is that the prevalence of NAFLD is increasing together with overweight/obesity, dyslipidemia, DM and hypertension, all components of MS. NAFLD is increased in individuals with glucose intolerance (43%) and those with recently diagnosed DM (62%). However, lean individuals with normal glucose tolerance may also develop the disease. The increasing prevalence of NAFLD and MS emerges as important public health issues [6,7,30–32].

Results of long-term studies with patients with HS and NASH had shown that NAFLD patients present higher overall mortality compared to the corresponding control populations. The most common cause of death in patients with NAFLD, NASH and HS is cardiovascular diseases, as they have common risk factors. Patients with NASH (but not with HS) have a higher mortality rate related to liver injury [5].

Most individuals with steatosis are asymptomatic. However, the most common characteristics involving liver damage markers are the two- to three-fold increase of serum levels of alanine aminotransferase (ALT) (cytoplasmic) and aspartate aminotransferase (AST) (cytoplasmic and mitochondrial) through cell lysis that occurs along with liver damage. The AST/ALT ratio is typically lower than one; when it is greater than one, disease progression is assumed, because at this moment cell and mitochondrial lysis occurs. Liver diseases with AST/ALT < 1 are often related to obesity, lack of exercise and hyperlipidemia, whereas AST/ALT > 1 is related with alcohol consumption and DM. High ALT levels in NAFLD suggest further development of DM [33,34].

Data published in the “Third National Health and Nutrition Survey” reflect significant association between high concentrations ALT and IR, type 2 DM and SM [35]. The mechanisms of action of insulin in the liver are different from those on adipose tissue and skeletal muscle; in the latter tissues, IR is a genetic or acquired disease where the physiological concentrations of the hormone do not sustain a normal response of glucose uptake in the cells. Lower glucose absorption increases insulin production by the pancreas to keep glucose levels normal, thus hyperinsulinemia is frequent in IR. In liver tissue, IR can be defined as a failure of the hormone to properly suppress hepatic glucose production. However, the lipogenic activity of insulin does not seem to be impaired in IR [36].

In IR, the activity of hormone-sensitive lipase is maintained (Figure 2) in peripheral tissues, increasing the supply of FFA to the plasma and subsequently to the liver, the body’s systemic damper. The intracellular increase of acyl-CoA and diacylglycerol (DAG) concentrations in extrahepatic tissues is associated with higher levels of TG and FFA, in addition to the decrease of the insulin receptor signaling pathway, which may in part induce IR. DAG is a metabolite of intracellular triglyceride hydrolysis and it is known as an allosteric activator of protein kinase C (PKC), which can phosphorylate the insulin receptor substrate 1 (IRS-1) at the serine/threonine site. As a consequence, IRS-1 phosphorylation is inhibited leading to lower binding of phosphatidylinositol 3-kinase to IRS-1 and lower translocation of glucose transporter (GLUT-4) to the membrane, stimulated by insulin, with subsequent worsening of hyperglycemia and hyperinsulinemia [4,13,37].

Some NAFLD mechanisms are under investigation, however, TG accumulation in hepatocytes as a result of IR is considered the first step to the current widely accepted pathogenic model. The OS resulting from mitochondrial oxidation of fatty acids and from the expression of inflammatory cytokines is considered a secondary causative factor to liver damage, fibrosis and inflammation [8,23,37].

In the liver, the action of several transcription factors related to the lipogenic and antilipolytic effects of insulin, including PPAR-γ (peroxisome proliferator-activated receptor), transcription factor sterol regulatory element binding protein (SREBP-1c) and ChREBP carbohydrate regulatory element-binding protein, remains unchanged even in states of IR [36], contributing to the accumulation of fat in hepatic tissue. Through the activation of nuclear receptors including the liver X receptors (LXRs), both LXR α and β have been shown to promote lipogenesis by activating SREBP-1c and its target genes. In addition to the LXR-SREBP pathway, accumulating evidence has suggested that LXRs have additional transcriptional targets, such as PPAR-γ, ChREBP, stearoyl CoA desaturase-1 (SCD-)1 and lipoprotein lipase (LPL), which may also contribute to the lipogenic effect [27].

It is widely believed that steatosis sensitizes the liver to secondary insults during the development of hepatic inflammation and other complications. The exact mechanism of the progression of HS to NASH is unknown. The most accepted hypothesis, the Day and James (1998) [38] hypothesis, divides the pathophysiological process in two stages. The first stage consists of the mechanism that causes HS, *i.e*., the initial onset. Thus, IR that results in hyperinsulinemia and favors lipolysis in adipose tissues, excessively increases the supply of FFA to the liver and may result in HS. The first stage leaves the hepatocyte more vulnerable to the second onset caused by viruses, alcohol and lipopolysaccharides, among others that promotes hepatic OS and leads to NASH, cirrhosis and hepatocellular carcinoma [29].

Another model by Wanless and Shiota, (2004) [39] proposes four stages for the progression of HS to more severe tissue damage. The first two stages comprise the previously described stages, the third is characterized by the leakage of lipids to the liver interstitium, leading to the direct damage and inflammation of hepatic veins. The fourth stage is characterized by venous obstruction with secondary collapse, fibrosis and cirrhosis [3,36]. NASH is the hepatic complication of the metabolic syndrome. The steatosis may induce a low-grade inflammatory response, similar to the adipose tissue inflammation that follows adipocyte lipid accumulation. Moreover, in obese individuals, hepatic inflammation and fibrosis may result from the exposure of the fatty liver to metabolic and pro-inflammatory mediators, produced by visceral fat and drained by the portal circulation [37].

The increased supply of fatty acids to hepatocytes increases mitochondrial beta-oxidation and the levels of cytochromes P450 and P450 2E1 (CYP2E1), leading to increased levels of ROS. OS has an important role in the transition of steatosis to steatohepatitis, although the exact molecular mechanism of NASH has not yet been elucidated. The accumulation of lipids in the liver with steatosis is associated with peroxidation, analogous to that observed in low density lipoproteins in atherosclerotic lesions. Thus, in NASH, the increased supply of FFA to the liver increases the generation of ROS as a result of electron loss in energy-producing reactions. Furthermore, the interaction AGEs/RAGE may subsequently alter gene expression in various types of cells including hepatocytes and hepatic stellate cells. The increase in the proliferation and activation the hepatic stellate cells participate in the progression to hepatic fibrosis in NASH [10,13,19,40,41].

## 3. AGEs: Formation and Effects

As already commented, AGEs are formed from non-enzymatic amino-carbonyl interactions. In these reactions, the reducing sugars or oxidized lipids react with proteins, amino acids, aminophospholipids or nucleic acids [14]. AGEs formation may occur in food subjected to high temperatures, such as frying and grilling, and in biological systems [42].

**Figure 3:**
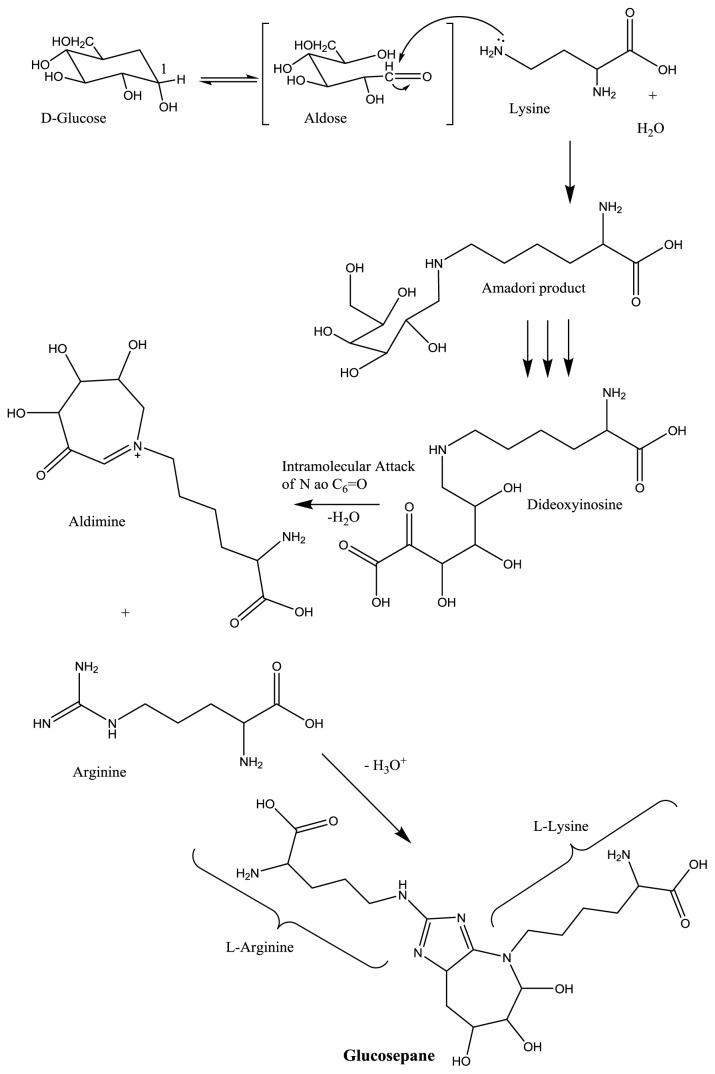
AGEs formation from reducing sugars. Nucleophilic attack on the anomeric carbon of the sugar by Lys present in proteins, forming Amadori product. Metabolic changes that may cause increased Amadori products are hyperglycemia (Maillard reaction), hipertriglyciridemia and increased fat deposits tissue (lipid peroxidation with formation of reactive carbonyl and dicarbonyl compounds), common processes in obese and type 2 diabetics. After successive displacements of the carbonyl group along the carbon skeleton of the sugar, the intermediate α-dicarbonyl dideoxyinosine is formed. The α-dicarbonyl intermediate suffers intramolecular nucleophilic attack on the carbonyl group by the ɛ*N*, giving aldimine, a precursor for plausible cross-linking agent Lys-Arg, glucosepane. Adapted from [9,17]. C-1, Anomeric carbon on sugar; ɛ*N*, terminal nitrogen of lysine; Lys, lysine; Arg, arginine.

The Maillard reaction, also known as glycation, is the most often described in the literature as a route for production of AGEs, however, it is noteworthy that these products can be formed through other pathways. Glycation starts with the formation of unstable Schiff bases generated by the condensation of the carbonyl group of a reducing sugar (glucose, fructose, galactose and ribose), intermediates of glucose metabolism (glucose-6-phosphate, fructose-6-phosphate, ribose-5-phosphate, deoxyribose-5-phosphate and glyceraldehyde), or polyol metabolites (fructose or fructose-3-phosphate) and dicarbonyl compounds originating from lipid peroxidation (glyoxal, MDA) with an amine group, originated, for instance, from the amino acid lysine (or other amino acids, nucleic acids and lipids) [9,10]. Subsequently, the Schiff base undergoes rearrangements that make this structure more stable, forming products known as Amadori products, which are initial products of the Maillard reaction. The Amadori products generated have reactive carbonyl groups that condense with primary amino groups, giving rise to AGEs (Figure 3) [14,43].

**Figure 4:**
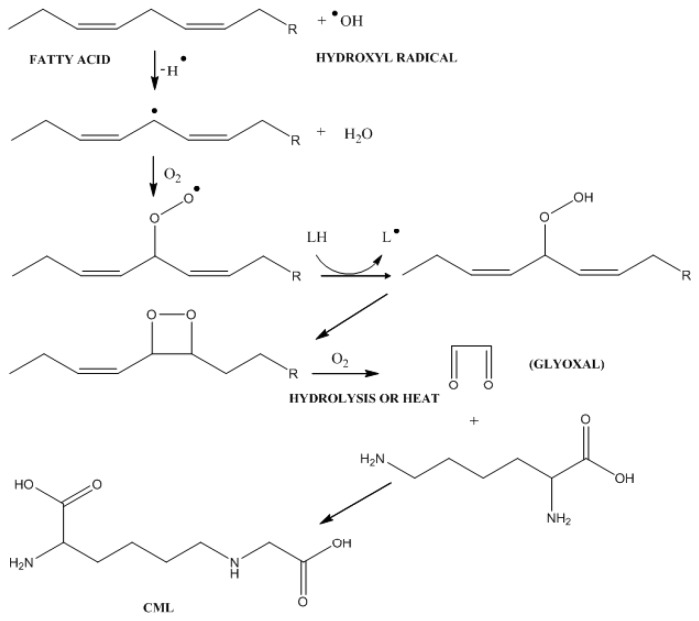
AGEs formation by oxidation of FFA. Hydroxyl radical abducts a hydrogen in the bis-allylic sp^3^ carbon of the conjugated lipid, forms a carbon radical and thereafter, in the presence of oxygen, a peroxyl radical that is reduced with another FFA to form a lipid peroxide, cyclizes and generates a cyclic peroxide, which in the presence of oxygen suffers breakage with formation of glyoxal, which reacts with lysine forming CML. ^•^OH, hydroxyl radical; H^•^, output hydrogen radical; LH, any lipid; L^•^, lipidic radical.

Alternatively, Amadori products may be fragmented by glycoxidation in the presence of ions of transition metals such as iron, resulting in the formation of short chain reactive factors such as glyoxal and methylglyoxal, which are also generated during accessory glycolytic stages or during LP. For diabetics and obese-diabetics, hyperglycemia is a factor that promotes greater production of Amadori products. Indeed, the formation of this product in obesity is more correlated with LP, which may also be present in obese-diabetics patients. This is an important difference between the pathophysiology of diabetes and obesity in the development of liver disease [9,14,16,22]. In NAFLD, the increased levels of OS and LP stimulate the formation of AGEs such as (carboxymethyl)lisine (CML), which is also characterized as a product of LP (Figure 4). Thus, it is likely that, in HS, this pathway is the most important for the generation of AGEs [15].

The increased production of Amadori products in the cellular environment could result in an early and irreversible damage, if there were no control mechanisms to limit their excessive formation. Among these mechanisms, serum concentrations of glucose and proteins, a proper control of cellular permeability to glucose mediated by insulin, and the reactivity of the amino groups of long and short-life proteins can be highlighted. Co-morbidities associated with IR and hyperglycemia, e.g., obesity and diabetes, result in an uncontrolled increase of Amadori products, contributing to the accelerated formation of AGEs [9,43].

The formation of AGEs was initially associated with the glycation of proteins with long half-life, however, especially in the presence of hyperglycemia, this process has been also observed in short half-life proteins. The most common sympton of aging at the molecular level is the accumulation both intra- and extra-cellularly of altered proteins. The accumulation of AGEs is related to the development of vascular, renal, retinal and liver damage and neural complications of diabetes [11,14,16].

Circulating AGEs consist of AGEs-modified plasma proteins, AGEs–low molecular weight compounds (for instance, AGEs peptides), and, the most abundant, AGEs free adducts (e.g., AGEs single amino acids) originating from exogenous AGEs and generated by direct modification of circulating amino acids or released during the degradation of AGEs-modified proteins and other macromolecules [9,14,16,17].

Removal of existing AGEs-crosslinks from tissue components is conducted largely through extracellular proteolysis and by scavenger cells such as tissue macrophages which ingest AGEs via AGEs-specific or nonspecific receptors. Generally, AGEs-modified molecules are recognized and internalized by cell-surface receptor mediated endocytosis, degraded intracellularly, and subsequently released as low molecular weight AGEs that include, reactive intermediates with high crosslinking or oxidative reactivity. These circulating AGEs can be eliminated by either renal or hepatic clearance. All clearance mechanisms of AGEs decrease with advancing age [9,14,17].

As previously mentioned, the pathological effects of AGEs are related to their ability to modify chemical and biological properties of native molecules [11,14]. The liver is a target tissue for the harmful effects of AGEs but it is also an important site for clearance and catabolism of circulating AGEs. The removal of senescent macromolecules is performed by several cell types such as macrophages, and Kupffer and LECS. In the case of AGEs, it has been reported that after intravenous administration of an AGE-modified bovine albumin prepared *in vitro*, over 90% of the glycated protein was cleared by Kupffer and liver sinusoidal cells. The mechanisms described above to AGEs is reduced in aging liver and hepatic disease, resulting in AGEs leakage into the circulation and in its extrahepatic deposition [18,44].

## 4. AGEs, RAGE and Increasing Oxidative Stress in Hepatic Disease

To act intracellularly AGEs need to bind to several receptors and ligand proteins such as RAGE, AGE-R1 (OST-48, p60), AGE-R2 (80KH phosphoprotein), AGE-R3 (galectin-3), the macrophage scavenger receptor ScR-II and CD-36. Unlike some receptors that frequently perform only AGEs scavenging and clearance from tissues and circulation, the membrane proteins RAGE mediate several cellular responses. RAGEs were first found in patients with diabetes, where their gradual buildup is responsible for the modulation of gene expression, and production of radicals and pro-inflammatory cytokines [16,17,44–46].

RAGE is a protein of approximately 45 kDa. It is a member of the cell surface immunoglobulin superfamily and its physiological expression is detected in a variety of tissues including endothelial cells, vascular smooth muscle, peripheral blood mononuclear cells and macrophages, neural tissue, lung and skeletal muscle. The ability to recognize several ligands is one of this molecule’s functional features, allowing its broad participation in pathophysiological events related mainly to the spread of cellular dysfunction. Upon increased cellular activity or stress, inflammation or Alzheimer’s disease, RAGE expression is increased in affected cells as a marker for inflammation [17,46,47].

AGEs/RAGE interaction may play beneficial or detrimental roles in regards to the activities and fate of AGEs. In the first case, some receptors may help in the removal of AGEs from the circulation and thus may lessen the pro-oxidant effects of AGEs. The truncated isoform of RAGE, e.g., soluble RAGE (sRAGE) and secretory RAGE, have been used as a powerful anti-inflammatory, acting as a trap for RAGE ligands [48]. In contrast, according to Assis *et al.* [13], the AGEs receptor and other receptors appear to activate a stress response that triggers inflammation and cellular dysfunction. Activation of RAGE may promote cell death or survival, depending on the cell type and the endogenous conditions. This dual function of RAGE is essential during development, when the control cell proliferation and apoptosis are required. In adulthood, their physiological expression is low, being increased by inflammatory mediators and accumulation of RAGE ligands, resulting in the amplification of free radical production and inflammation [46].

The sRAGE circulates in human plasma and has emerged as a reliable biomarker in a number of RAGE-mediated disorders. Of note, levels of sRAGE have been recently linked to IR and several components of the metabolic syndrome. In Yilmaz *et al.* [49], which aimed to examine the relationship among plasma sRAGE levels, IR, and biochemical and histological parameters of liver injury in a cohort of NAFLD patients and a pro-inflammatory state, it was observed that the concentrations of sRAGE were significantly lower in patients with definite NASH and borderline NASH compared to control subjects. No significant differences were found in patients with simple fatty liver as compared to controls. These findings suggest that lower concentrations of sRAGE are associated with the most severe forms of NAFLD. Evidence has suggested that sRAGE may act as a decoy receptor of AGEs and other pro-inflammatory ligands, thereby preventing AGE from binding to the cell-bound full-length receptor RAGE [50,51].

**Figure 5:**
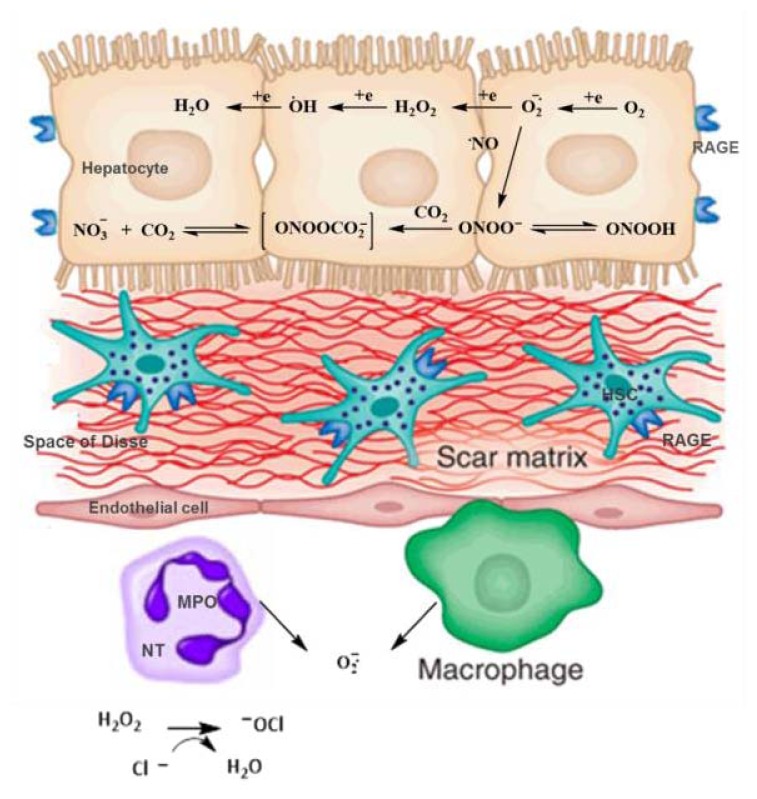
Oxidative stress and expression of RAGE in the liver. ROS formation by one-electron reduction step of O_2_. O_2_^•−^ is formed largely through mitochondrial respiration. During spontaneous dismutation of O_2_^•−^, H_2_O_2_ is formed. H_2_O_2_ can also be formed in peroxysomes. If NO is present, O_2_^•−^ will react to form ONOO^−^ and ONOOH. The peroxynitrite can then react with carbon dioxide to generate downstream nitrating species. In the extracellular fluid, Kupffer cells and neutrophiles are sources of O_2_^•−^, and neutrophiles release the enzyme MPO to produce OCl^−^ from hydrogen peroxide and chloride. The presence of RAGE in hepatocytes and HSCs increases hepatic oxidative stress. KC, Kupffer cells; NT, neutrophile; HSC, HSCs; MPO, myeloperoxidase; RAGE, receptors for AGEs (Adapted from Jaeschke, H. & Ramachandran A. 2011 [2]; Friedman S.L. 2008 [52]).

In the liver, there seems to be a specific pattern for RAGE expression, with little or no expression in Kupffer cells and LECS, and expression in hepatocytes and HSCs, as observed in studies with humans and rats. The potential role of RAGE in hepatic diseases or its protective effect has been the subject of several studies. *In vitro* research and studies with animals show that the axis ligand/RAGE plays a role in the activation of intracellular signaling, resulting in the production of cytokines that may be associated with inflammation processes or tissue regeneration (Figure 5) [11].

Gaens *et al.* [15], studying liver biopsy and serum samples from obese individuals, observed that the immunohistochemical staining of RAGE in liver biopsies was specifically and exclusively located in the membrane of steatotic hepatocytes, whereas non-steatotic hepatocytes showed no staining for RAGE. The accumulation of CML and the formation of CML and proinflammatory cytokines expression during intracellular lipid deposition in *in vitro* cultured hepatocyte were also studied. The *in vitro* study showed that the accumulation of lipids in hepatocytes was associated with increased formation of CML and increased expression of RAGE, plasminogen activator inhibitor-1 (PAI-1), interleukin 8 (IL-8), interleukin 6 (IL-6) and C-reactive protein (CRP). These results show that the development of steatosis is associated with AGEs accumulation and that CML may have a subsequent role in inducing liver inflammation through increased RAGE expression.

Recent results show that OS, late inflammatory responses in several types of endothelial cells, activation of LECS involved in fibrogenesis, increased expression of C-reactive protein, and induction of IR are among the roles of AGEs through its interaction with RAGE, suggesting a critical role for these molecules in liver damage [16,18]. Data indicate that AGEs may cause damage to the β-cells of the islets of Langerhans in the pancreas and peripheric IR, which may lead to the initiation and progression of previously unforeseen pre-diabetes to diabetes [1,53–55].

An *in vivo* study confirmed that rats fed with a diet rich in AGEs showed reduced levels of reduced glutathione/oxidized glutathione ratio and higher concentrations of 8-isoprostanes (a lipid peroxidation product) than animals fed with a diet poor in AGEs [55]. The work of Patel *et al*. [19] reinforces the hypothesis that the AGEs/RAGE interaction may lead to OS and hepatic inflammation with subsequent disease progression independent of steatosis. It was shown that focal hepatic inflammation was detected in rats with 26 weeks of age fed on a diet rich in AGEs, while animals fed with a diet containing a regular amount of AGEs remained without inflammation until the end of the experiment (39 weeks). This is consistent with the well-established concept that dietary AGEs may initiate oxidative stress through AGEs receptors expressed in liver cells. The fact that steatosis is not a feature of liver with inflammation suggests that AGEs from diet alone is sufficient to cause hepatic inflammation, independent of steatosis.

## 5. The Role of ROS and AGEs in the Evolution of NAFLD

The mechanisms for generation of ROS typically occur in the mitochondria, cell membranes and cytoplasm. For generations of ROS, the mitochondria is the site of larger production. In this organelle, the reaction takes place in the mitochondrial electron transport chain through cytochrome oxidases, being the main source of reactive species due to the univalent reduction of O_2_, generating superoxide anion radical (O_2_^•−^), hydroxyl radical (OH•) and hydrogen peroxide (H_2_O_2_) [21,56].

The oxidation reactions in the mitochondria (beta-oxidation, citric acid cycle) are associated with the conversion of oxidized cofactors (NAD^+^ and FAD) into reduced cofactors (NADH and FADH_2_). The major sites for the physiological production of ROS generation are the complexes I and III of the mitochondrial electron transport chain. During increased energy supply, the ratio NADH/NAD^+^, the reduction level of complex I, and ROS production are high. In IR, a common feature of NAFLD, the flux of FFA into the circulation due to the lipolysis in adipocytes is exacerbated, resulting in increased mitochondrial oxidation of these substrates in the liver and increased generation of ROS, since it shares common steps with glucose oxidation [9,24,56].

Studies with animals and humans show that OS plays an important role in the pathogenesis of NAFLD/NASH [57]. In the absence of alcohol intake, patients presenting MS or any of its components involving IR, develop hepatic steatosis due to enhanced lipolysis and increased FFA delivery from adipose tissue to the liver (Figure 2). In this case, hepatic tissue becomes more susceptible to OS due to the abnormal accumulation of fat in the cells, which may lead to the second stage of NAFLD and the progression of simple steatosis to steatohepatitis and fibrosis, characterized by inflammation [41].

**Figure 6:**
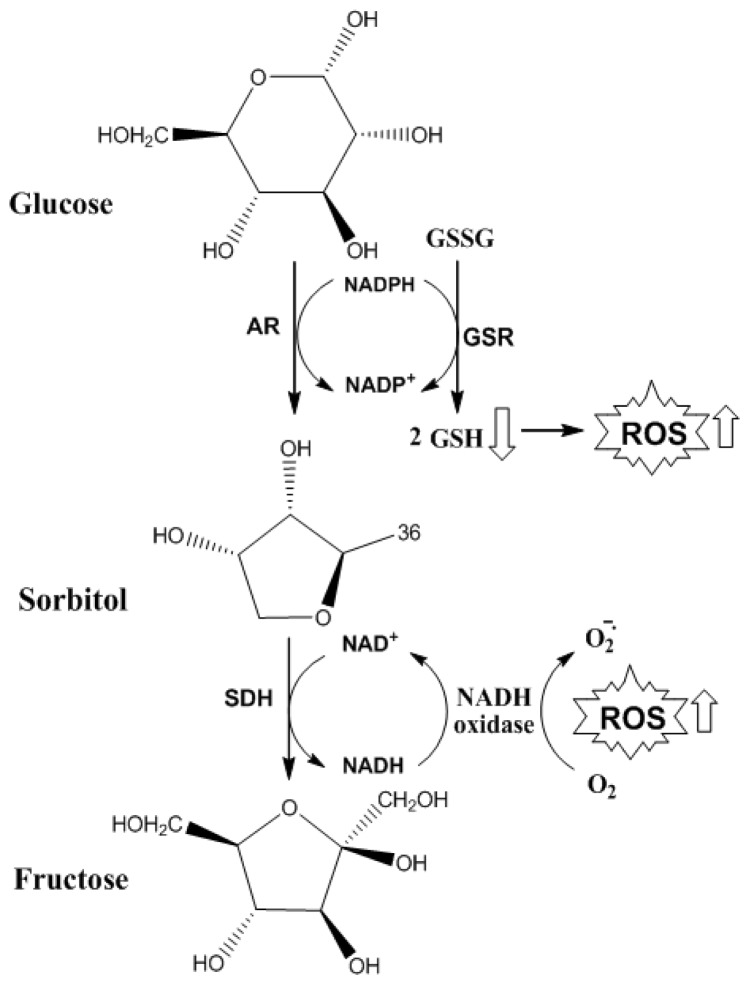
Pathways for the formation of polyol and ROS. Glucose in the presence of AR and NADPH is reduced, forming sorbitol and NADP^+^; then sorbitol is oxidized in the presence of SDH and NAD^+^, forming fructose and NADH. During the reduction of glucose to sorbitol, together, oxidized glutathione is reduced to two molecules of glutathione by glutathione reductase in the presence of NADPH. In conditions of larger amounts of glucose to sorbitol reduction, lower levels of NADPH are available for the reduction reaction of oxidized glutathione to glutatione, with an increase of ROS. In higher oxidation of sorbitol to fructose, there will be a higher availability of NADH that in the presence of NADH oxidase and oxygen, increases the release of superoxide anion radical, with consequent increase of ROS release. AR, aldose reductase; NADPH, reduced nicotinamide adenine dinucleotide phosphate; NADP^+^, oxidized nicotinamide adenine dinucleotide phosphate; GSSG, oxidized glutathione; GSR, glutathione reductase; GSH, reduced glutathione; ROS, reactive oxygen species; NAD^+^, oxidized nicotinamide adenine dinucleotide; SDH, sorbitol dehydrogenase; NADH, reduced nicotinamide adenine dinucleotide; NADH oxidase, nicotinamide adenine dinucleotide oxidase.

As described in the present work, LP is important for ROS and AGEs generation; however, it is noteworthy that IR and hyperglycemia may also be present in patients with NAFLD, contributing to the increased lipid deposition by *de novo* lipogenesis. Thus, hyperglycemia may induce OS through the mechanisms described above, by direct formation of AGEs, or by activation of polyol pathways (Figure 6). In the polyol stage, aldose reductase uses NADPH to reduce glucose to sorbitol. Under normal conditions, sorbitol production by aldose reductase is a less active reaction. However, under conditions of hyperglycemia approximately 30 to 35% of glucose is metabolized in this stage. At this time, NADPH availability is reduced, thus decreasing the regeneration of glutathione and NOS synthase activity, increasing OS. In another stage of the reaction, the second enzyme sorbitol dehydrogenase oxidizes sorbitol into fructose with concomitant production of NADH. Upon NADH increase, NADH oxidases produce superoxide anion radical. Furthermore, they may also induce the production of mitochondrial superoxide anion radical (Figure 6) [57–59].

In the metabolism of sorbitol to fructose, fructose-1-phosphate is produced with subsequent formation of glyceraldehyde and dihydroxyacetone from its breakage. Glyceraldehyde may be metabolized into glyceraldehyde-3-phosphate and thus return to glycolysis, or react with amino groups and thus participate in AGEs formation. Yet dihydroxyacetone produces phosphatidic acid and diglycerides, where the latter are also involved in AGEs production [9,57–59].

**Figure 7:**
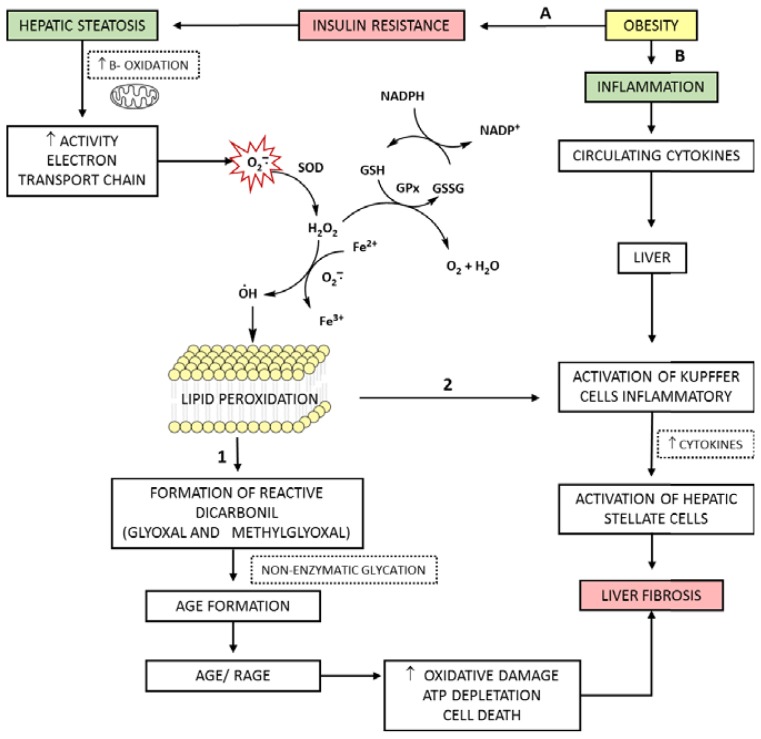
Evolution of hepatic steatosis to steatohepatitis: Role of AGEs and oxidative stress—a vicious cycle. Obesity is associated with IR (**A**) and inflammation (**B**). (**A**): The IR triggers metabolic changes responsible for the development of hepatic steatosis. Fat oxidation is increased, with higher beta-oxidation reaction and mitochondrial activity. The increased activity in the electron transport chain leads to mitochondrial dysfunction with subsequent formation of ROS; the ^•^O_2_^−^ formed can be reduced by enzymatic systems to H_2_O_2_ (SOD) and by subsequent reaction (GPx) to water; however its reaction with transition metals such as Fe^2+^ can lead to the formation of the ^•^OH, with subsequent attack to the cell membrane, resulting in lipid peroxidation (LP) in intracellular organelles. Lipid peroxidation: (**1**) Source of reactive dicarbonyl compounds (reaction intermediates of glycation) which form AGEs (**2**) Trigger the activation of the inflammatory system, and spread via increased OS. Interaction AGEs/RAGE increases ROS formation, these mechanisms lead the damage oxidative, ATP depletion, cell death, fibrosis and progression of liver disease; (**B**) Inflammatory cytokines produced by adipose tissue are directed to the liver, strengthening the activation of Kupffer cells inflammatory together the ROS with activation of hepatic stellate cells, contributed to the worsening of fibrosis. SOD, superoxide dismutase; GPx, Glutathione peroxidase; GSH, reduced glutathione; GSSG, oxidized glutathione; NADP^+^, oxidized nicotinamide adenine dinucleotide phosphate; NADPH, reduced nicotinamide adenine dinucleotide phosphate; Fe^2+^, ferrous iron; Fe^3+^, ferric iron; AGEs, advanced glycation end products; RAGE, receptor of advanced glycation end products; ATP, adenosine triphosphate; TNF-α, tumor necrosis factor alpha; IL-6, Interleukin 6; IL-8, Interleukin 8.

Associated diseases, such as MS and aging, have a pathogenesis related to OS from mitochondrial damage. Damaged mitochondria produces lower amounts of adenosine triphosphate and higher amounts of ROS, leading to OS and to a lower usage of carbohydrates and lipids. These unused macronutrients accumulate outside the mitochondria, amplifying the process of glycation, AGEs production, and related diseases [20,24]. The AGEs/RAGE interaction results in intracellular generation of OS and subsequently in the activation of transcription factors such as NF-κB, which leads to a greater inflammatory response and local tissue damage [49,59]. OS also stimulates AGEs/RAGE interaction, causing an increase of AGEs-dependent RAGE, and thus a vicious circle of AGEs/RAGE appears, where OS is initiated and accelerated (Figure 7) [48].

Other consequences of increased OS include inflammatory hepatic system and systemic activation. The inflammation accompanies the majority of acute and chronic liver disorders and hepatotoxic conditions. In this condition, liver-resident immune cells (for instance, Kupffer cells, HSCs, LSCs) activate the adaptive responses, in an attempt to cope with stress. In this step, cells that are recruited in response to injury (for instance, monocytes, macrophages, dendritic cells, natural killer cells) emit pro-inflammatory signals including cytokines (TNF-α, IL-6 and IL-1), chemokines, lipid messengers, and reactive oxygen species. This generally results in mild inflammation, contributing to the re-establishment of homeostasis. However, when such adaptive responses fail, stressed hepatocytes can suffer apoptosis or necrosis, aggravating the inflammatory response [60–62]. Such a response can lead to fibrosis process, where Kupffer cells (and other liver cells) stimulate the enlarged hepatic population of HSCs, which are responsible by progressive accumulation of scarring proteins due to an imbalance between fibrogenesis and fibrolysis (Figure 7) [60–62].

## 6. Final Considerations

Diseases with pathophysiology associated to IR such as diabetes, hypertension and dyslipidemia, present metabolic characteristics common to NAFLD and have OS as a main factor in their aggravation. One can observe that both redox dysfunction and non-enzymatic glycation are able to increase hepatic inflammation, where each one starts the reaction in a specific manner. In non-enzymatic glycation, proteins (from serum or cell) accumulate successive glycation processes with subsequent loss of function and initiation of clearing and repairing reactions that, on occasion, may trigger an inflammatory process. Further, the formed AGEs may interact with specific cellular receptors and lead to chain reactions that increase intracellular OS and inflammation. All these reactions are commonly found in hyperglycemic and/or diabetic individuals. However, in overweight or obese and/or NAFLD carriers, besides the mentioned hyperglycemia and damage, a combined or isolated increase of the lipid supply to the liver, either by the increased delivery of fatty acids from adipose tissue or by the abnormal accumulation in the tissue, may occur. These individuals are more susceptible to abnormalities in mitochondrial function followed by lipid peroxidation in hepatocytes. The increase in the inflammatory process may be activated directly by ROS and/or by AGEs, as lipid peroxidation intermediates are able to continue the stages of non-enzymatic glycation, as is the case of CML. The cumulative effects of such events combined with genetic predisposition and environmental conditions may lead to the progression of hepatic dysfunction into later stages of the disease that are difficult to reverse and to treat.

## 7. Conclusions

Functionally, the liver is the body’s systemic shock absorber. Hepatic steatosis may be considered as a response or adaptation to molecular mechanisms related to overweight/obesity, IR and hyperglycemia. In the present work, it was possible to observe that NAFLD share common cellular dysfunctions with chronic degenerative diseases which are widely studied under the latter condition, but not in hepatic disease. The study of the combined activity of OS and AGEs by sharing metabolic pathways, substrates or products presented in this review are crucial to understand the pathophysiology of NAFLD and can be used to devise rational therapeutic interventions for this disease, being OS a key process in development of complications. The traditional treatment for NAFLD is restricted to the stimulation of physical activity and weight loss. Experimental studies using antioxidants and antiglycants in nutritional and pharmacological therapies have been evaluated. However, despite the benefits observed, their specific prescription is not yet available to the population. Thus, a greater involvement of the scientific community is necessary for the establishment of novel therapies, as NAFLD follows obesity, and are diseases in rise.

## Figures and Tables

**Table 1:** Diseases: description and diagnostic criteria.

Disease	Description	Diagnosis	Ref.
Metabolic Syndrome (MS)	Complex disorder represented by a set of cardiovascular risk factors, usually related to central fat deposition and IR	Abdominal obesity, defined by increased waist circumference (≥90 cm in men and ≥80 cm in women) and two or more of the following characteristics: blood pressure (≥130/85 mm/Hg), high levels of fasting glucose (≥100 mg/dL) or triglycerides (≥150 mg/dL) or low levels of HDL cholesterol (<40 mg/dL in men, <50 mg/dL in women)	[5–8]
Diabetes	Heterogeneous group of metabolic disorders that have in common the hyperglycemia, which is defective in insulin action, insulin secretion, or both	World Health Organization (WHO) criteria: fasting plasma glucose (FPG) ≥7.0 mmol/L (126 mg/dL) or 75 g oral glucose tolerance test (OGTT) with FPG ≥ 7.0 mmol/L (126 mg/dL) and/or 2 h plasma glucose ≥ 11.1 mmol/L (200 mg/dL) or, glycated haemoglobin (HbA1c) ≥6.5% /48 mmol/mol, or Random plasma glucose ≥11.1 mmol/L (200 mg/dL) in the presence of classical diabetes symptoms.	[5,6,8,9]
Nonalcoholic fatty liver disease (NAFLD)	Encompasses the entire spectra of fatty liver diseases in individuals without significant alcohol consumption, ranging from fatty liver to steatohepatitis and cirrhosis	(a) presence of hepatic steatosis by imaging or histology; (b) no significant alcohol consumption; (c) no competing etiologies for hepatic steatosis; and (d) no co-existing causes for chronic liver disease.	[5,7,10]
Hepatic Steatosis (HS)	Presence of hepatic steatosis with no evidence of hepatocellular injury in the form of ballooning of the hepatocytes or no evidence of fibrosis	Liver biopsy with histological analysis	[5]
Nonalcoholic Steatohepatiti s (NASH)	Presence of hepatic steatosis and inflammation with hepatocyte injury (ballooning) with or without fibrosis	Liver biopsy with histological analysis NAFLD Fibrosis Score *	[5]
Hepatic Cirrhosis	Presence of cirrhosis with no obvious etiology. Patients with cryptogenic cirrhosis are heavily enriched with metabolic risk factors such as obesity and metabolic syndrome	Liver biopsy with histological analysis NAFLD Fibrosis Score *	[5]

* Clinically useful tools for identifying NAFLD patients with higher likelihood of having bridging fibrosis and/or cirrhosis. NAFLD Fibrosis Score is based on six readily available variables (age, BMI, hyperglycemia, platelet count, albumin, AST/ALT ratio) and is calculated using the published formula (http://nafldscore.com) [5].
